# Quantitative CT lung densitometry as an obstructive marker for the diagnosis of bronchiolitis obliterans in children

**DOI:** 10.1371/journal.pone.0271135

**Published:** 2022-07-07

**Authors:** Hye Jin Lee, Seong Koo Kim, Jae Wook Lee, Soo Ah Im, Nack-Gyun Chung, Bin Cho

**Affiliations:** Department of Pediatrics, College of Medicine, The Catholic University of Korea, Seoul, Republic of Korea; Clinic for Infectious and tropical diseases, Clinical centre of Serbia, SERBIA

## Abstract

The purpose of this study is to evaluate the quantitative diagnostic performance of computed tomography (CT) densitometry in pediatric patients with bronchiolitis obliterans (BO). We measured the mean lung density (MLD) and represented the difference of MLD in inspiratory and expiratory phases (MLDD), the ratio of the MLD (E/I MLD), and the relative volume percentage of lung density at 50-Hounsfield unit (HU) interval threshold (E600 to E950). We calculated the sensitivity, specificity, and diagnostic accuracy of the lung density indices for the diagnosis of BO. A total of 81 patients, including 51 patients with BO and 30 controls, were included in this study. In the BO patients, expiratory (EXP) MLD and MLDD were significantly lower, and E/I MLD and expiratory low attenuation areas below the threshold of −850 HU to −950 HU (E850, E900, and E950) were statistically significantly higher than controls. Multivariate logistic regression analysis showed that MLDD (odds ratio [OR] = 0.98, p < .001), E/I MLD (OR = 1.39, p < .001), and E850 to E950 were significant densitometry parameters for BO diagnosis. In a receiver-operating characteristic analysis, E900 (cutoff, 1.4%; AUC = 0.920), E/I MLD (cutoff, 0.87; AUC = 0.887), and MLDD (cutoff, 109 HU; AUC = 0.867) showed high accuracy for the diagnosis of BO. In conclusion, the lung CT densitometry can serve as a quantitative marker providing additional indications of expiratory airflow limitation in pediatric patients with BO.

## Introduction

Bronchiolitis obliterans (BO) is a chronic obstructive lung disease that leads to the irreversible obliteration of the small airways [1, 2]. BO can occur with various etiological factors, including infection (post-infectious BO [PIBO]), allogeneic hematopoietic stem cell transplantation (HSCT), and organ transplantation [3]. PIBO occurs more frequently in children than adults, and adenoviruses, influenza, respiratory syncytial virus, and mycoplasma pneumonia have been shown to be associated with PIBO [4]. Post-HSCT BO is a subtype of chronic lung graft versus host disease (GVHD), and the incidence reportedly varies from 1.7% to 26% [5]. The major histopathological mechanism of BO is fibrosis and compensatory overgrowth of tissues due to chronic inflammation of bronchial epithelial and sub-epithelial cells, which leads to terminal airway obstruction and stenosis [6].

Early diagnosis of BO in young children is particularly important, as it affects their long-term pulmonary function. The diagnosis of BO is generally based on a combination of clinical features and radiological findings, although lung biopsy and histopathological examination remain the diagnostic gold standard. The common computed tomography (CT) findings in patients with BO include bronchiectasis, bronchial wall thickening, and air trapping [7]. Conventionally, spirometry and chest CT findings have been suggested as the two most important standards for the diagnosis of BO. However, diagnosis of early BO is often challenging because CT changes such as air trapping and bronchial wall thickening can be subtle in the initial stages of BO. In younger children, this is further compounded by the inability to perform accurate pulmonary function tests (PFTs). Hence, development of additional tools that can improve the diagnosis of BO is desirable, particularly in younger children.

The purpose of this study was to evaluate the quantitative diagnostic performance of CT in pediatric patients with BO by analyzing various lung density indices (LDIs) based on mean lung density (MLD) measured during inspiration and exhalation using the chest CT quantitative method.

## Subjects and methods

### Study population

We performed a retrospective chart review of all pediatric patients under the age of 18 who underwent three-dimensional (3-D) chest CT at the Department of Pediatrics, Seoul St. Mary’s Hospital from March 2019 to March 2021. The inclusion criteria for this study were those who performed 3-D chest CT that met the following criteria; (a) patients with BO confirmed by a pediatric pulmonologist (n = 51) or (b) for regular evaluation before hematopoietic stem cell transplantation without prior lung disease (n = 16) or (c) evaluation for other chest symptoms without concomitant pulmonary disease (suspicious abnormal shadows on plain chest X-rays at regular health examination, later confirmed as normal lungs on chest CT in 8, physiological chest pain or costochondritis 3, physician-diagnosed gastroesophageal reflux disease 2, and screening for fire smoke inhalation injury 1, total n = 14, Table 1). Both (b) and (c) were included in the control group. The exclusion criteria for patients included in this study were as follows; (a) obstructive pulmonary disease other than BO (e.g., asthma or chronic lung disease of prematurity, n = 26) (b) patients under 3 years of age who do not cooperate well with inhalation and exhalation instructions (n = 2, aged 2 and 3 years). All BO patients were diagnosed by a pediatric pulmonologist based on clinical and radiological evidence. Clinical findings included 1) obstructive lung disease with a forced expiratory volume in 1 second (FEV1) or FEV1-to-forced vital capacity (FVC) ratio < 75% or 2) auscultation of expiratory rhonchi or wheezing on physical examination. BO was diagnosed when radiological findings such as air trapping, mosaic attenuation, bronchial wall thickening, or bronchiectasis were accompanied by appropriate clinical symptoms. Chest CT lung densitometry, spirometry, and body plethysmography were performed with written informed consent from the parent or legal guardian of each patient. This study was approved by the institutional review board of Seoul St. Mary’s Hospital (IRB number: KC21RASI0425).

**Table 1:** Patient characteristics in all patients.

	Control (N = 30)	BO (N = 51)	*p*-value
Median age (IQR), years	12.5 (9–15)	9.0 (6–15)	0.158
Gender, male	18 (60.0%)	32 (62.7%)	0.993
Diagnosis	−	Post-HSCT BO, 44 (86.3%) PIBO, 7 (13.7%)	< .001
FVC %	95.3 ± 12.2	73.7 ± 23.3	< .001
FEV1%	88.4 ± 12.3	53.2 ± 21.8	< .001
FEV1/FVC %	86.2 ± 6.0	66.7 ± 18.8	< .001
FEF25-75%	86.1 ± 19.3	36.6 ± 32.1	< .001
TLC %	95.0 ± 10.8	99.5 ± 24.4	0.358
RV %	91.0 ± 32.6	172.8 ± 84.0	< .001
DLCO %	77.6 ± 15.4	67.0 ± 21.2	0.048
VA L	3.6 ± 1.1	2.5 ± 1.0	< .001
sRaw %	134.5 ± 48.9	277.4 ± 253.0	0.004
INS V mL	2868.4 ± 1367.1	2393.3 ± 1308.7	0.125
EXP V mL	1558.2 ± 761.4	1674.9 ± 823.4	0.531
INS MLD, HU	-788.0 ± 74.5	-794.6 ± 71.0	0.689
EXP MLD, HU	-659.0 ± 61.6	-731.4 ± 69.6	< .001
MLDD, HU	129.0 ± 54.3	63.8 ± 47.2	< .001
E/I Volume %	57.6 ± 15.0	73.3 ± 18.3	< .001
E/I MLD %	83.9 ± 6.2	92.2 ± 5.9	< .001
E950%	0.0 ± 0.0	0.8 ± 1.7	0.002
E900%	0.4 ± 1.0	7.7 ± 11.3	< .001
E850%	3.9 ± 9.7	22.6 ± 21.6	< .001
E650%	20.7 ± 10.2	10.9 ± 7.5	< .001
E600%	26.2 ± 13.8	13.8 ± 9.2	< .001

Definition of abbreviations: BO, bronchiolitis obliterans; HSCT, hematopoietic stem cell transplantation; PIBO, post infectious BO; INS, inspiratory; EXP, expiratory; MLD, mean lung density; MLDD, mean lung density difference; HU, Hounsfield units; E950, expiratory low attenuation volume percentage under 950 HU.

### CT and PFTs

Prior to undergoing the CT scan, all the patients received instructions and training from experienced radiologic paramedics to achieve full-inspiratory and end-expiratory breath holds. We performed the CT scan without anesthesia or sedation on patients who were able to cooperate and for whom we were able to obtain full inspiration and expiration images. However, for patients who were younger than six years of age and uncooperative, chloral hydrate sedation was performed as needed. A 2-phase CT scan was performed by matching the timing of the visual full-inspiratory and expiratory phases according to the patient’s respiratory cycle. CT scans during full inspiration and end expiration were obtained craniocaudally in the supine position throughout the entire thorax without intravenous contrast material. All CT examinations were performed using a helical CT scanner (Somatom Force, Siemens Medical Systems, Erlangen, Germany) with a 250 to 350 mm field of view, 512 × 512 reconstruction matrix, 80–100 kVp, effective mAs (CARE Dose4D), and a tube rotation time of 0.25 ms. For lung parenchymal analysis, the images obtained were reconstructed using the following parameters: 1-mm thickness, no interval, and Br36 kernel. CT attenuation measurements were taken using the CT Pulmonology software application in a 3D solution program (Syngovia, Siemens). The application calculated the attenuation coefficient for each voxel in the lung and the frequency distribution of the voxels with specific attenuation numbers. We used iterative reconstruction in the CT images we analyzed [8].

In the PFTs, FVC, FEV1, FEV1/FVC, forced expiratory flow between 25% and 75% (FEF25–75%), residual volume (RV), total lung capacity (TLC), diffusing capacity for carbon monoxide (DLCO), alveolar volume (VA), and specific airway resistance (sRaw) were measured. All PFTs were performed with a Vmax instrument (Sensor Medics, VIASYS Healthcare, Yorba Linda, CA) in accordance with the guidelines of the American Thoracic Society and European Respiratory Society.

### Lung density index

**Fig 1:**
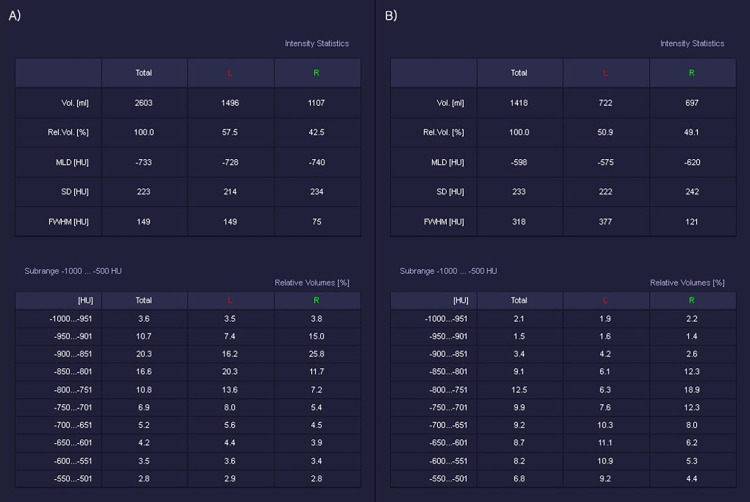
Example of densitometry parameter measurements using the 3D chest CT of a representative subject in the A) inspiratory and B) expiratory phases.

For each side of the lungs, separate inspiratory and expiratory lung volumes and densities were measured. We obtained the MLD during inspiration and expiration, and the ratios of end-expiratory to inspiratory volume (E/I volume) and MLD (E/I MLD) were calculated. To investigate the threshold of pathologic expiratory airflow limitation, we examined the relative volume percentage between −850 and −950 HU, with an interval of 50 HU. Expiratory low attenuation areas (LAAs) below the threshold of −850 HU to −950 HU (E850, E900, and E950) and high attenuation areas (HAAs) above the threshold of −600 HU to −650 HU (E600 and E650) were measured. Fig 1 shows an example of the CT results obtained from a representative subject.

### Statistical analysis

Descriptive analyses of qualitative variables are expressed as number of patients for each category and percentage. Quantitative variables are presented as mean and standard deviation for normally distributed variables or as median and interquartile range (IQR) when not. Comparisons between patients with BO and control group were done using Chi-square test for categorical variables and Mann-Whitney test for continuous variables. Correlations between the various quantitative lung density indices and the PFT parameters were compared using Spearman’s rho (*ρ*) correlation coefficient. We also tested the associations between the LDIs and PFT results using linear regression models, with adjustments for age and sex. Using multivariate logistic regression, we analyzed the odds ratio (OR) of the diagnosis of BO based on lung density and PFT parameters. Stepwise backward elimination and all subset regressions were performed to select the final model in the multivariate analysis. The receiver-operating characteristic (ROC) method was used to evaluate the usefulness of the lung density parameters for predicting physician-diagnosed BO. Areas under the curve (AUC) and optimal cutoff points based on maximizing the sum of the sensitivity and specificity were calculated for each densitometry parameter. All *p*-values are two-sided and *p* < 0.05 were considered statistically significant unless otherwise stated. All statistical analyses were performed using R Version 4.0.5 (https://cran.r-project.org/web/packages/maxstat/index.html).

## Results

### Patients’ characteristics

The demographic and clinical characteristics of the patients are presented in Table 1. A total of 81 patients including 30 controls and 51 patients with BO, were included in the study. Of the 51 patients with BO, 44 (86.3%) who previously received HSCT and 7 with PIBO (13.7%) were included. Spirometry and body plethysmography were performed in 62/81 (76.5%) and 56/81 (69.1%) of all patients, respectively. Spirometry was performed in 24/30 (80.0%) of the controls, 38/51 (74.5%) of the BO patients. Plethysmography was performed in 24/30 controls (80%) and 32/51 patients with BO (62.7%). Those who failed to undergo the PFTs were either too young or unable to do forced inhalation and exhalation because of severe BO. 32/51 (62.7%) of BO patients were on systemic steroids for previously diagnosed BO or other GVHD.

**Fig 2:**
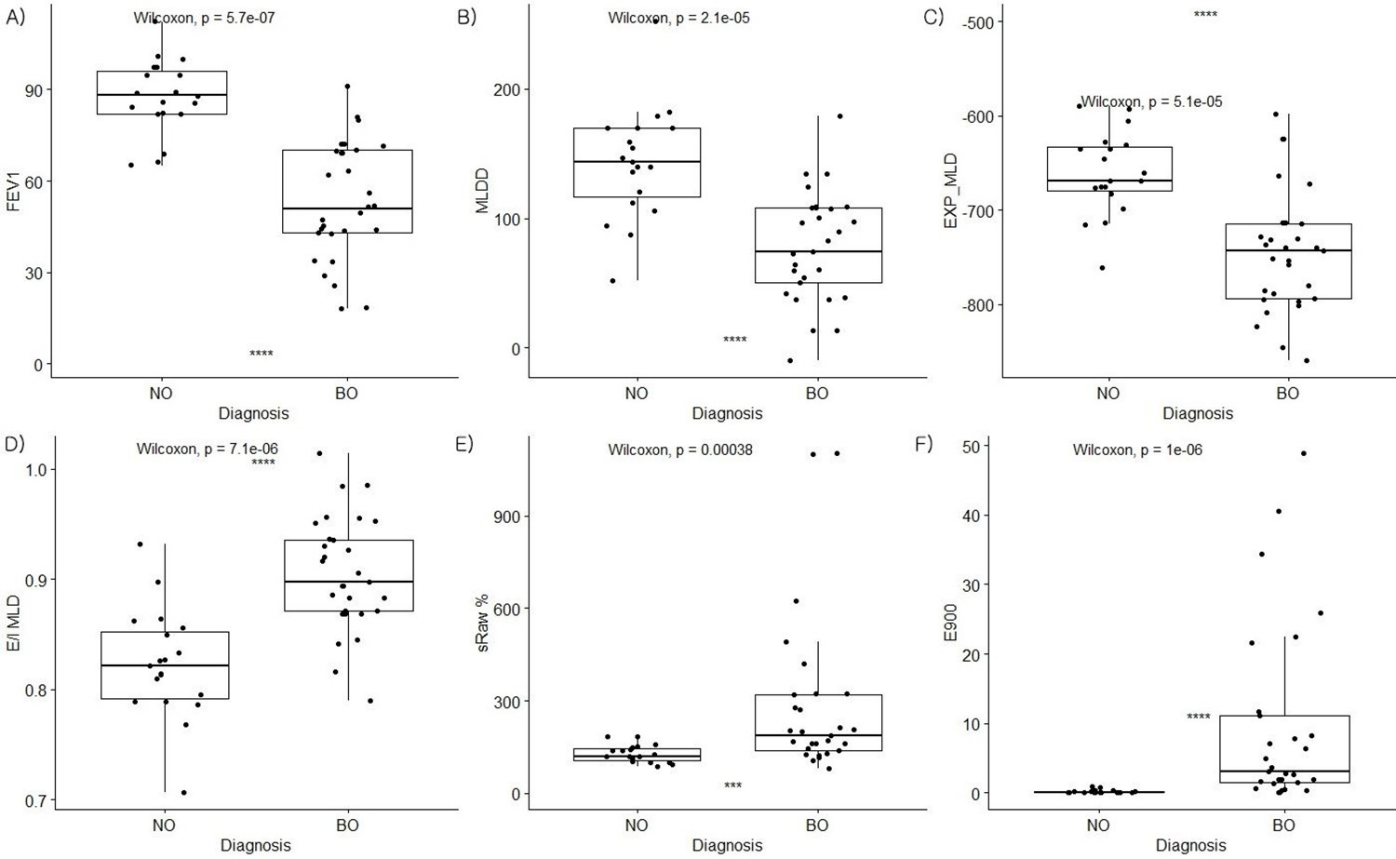
Comparison of quantitative lung densitometry indices and conventional pulmonary function test results according to diagnosis.

**Fig 3:**
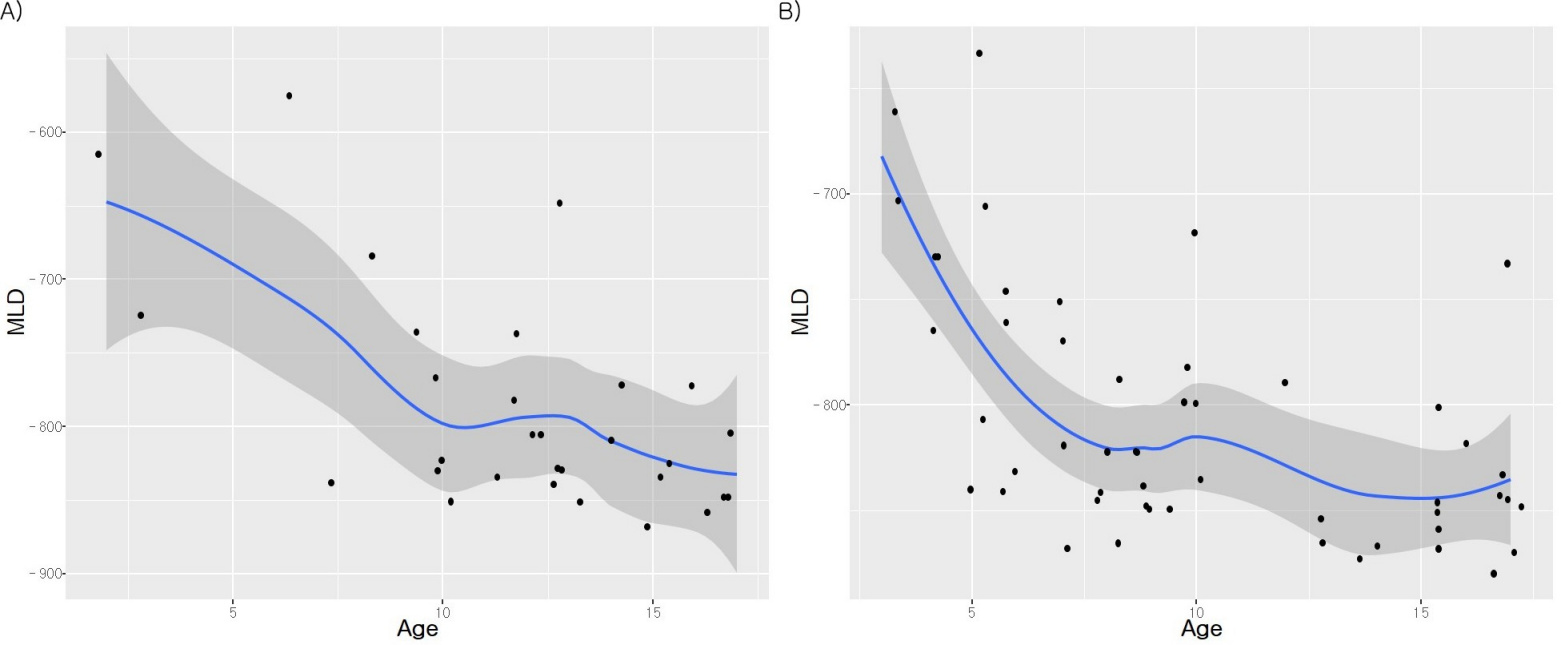
Scatter plot of inspiratory mean lung densities according to age for the A) control and B) bronchiolitis obliterans (BO) groups.

The median age was 12.5 (IQR, 9–15) years in the control group and 9.0 (IQR, 6–15) years in the BO patients. We reviewed the radiologist reports of the X-rays and CT results of all patients with BO. The X-ray images showed prominent bronchial markings and peribronchial thickening in 13, bronchiectasis 2, interstitial thickening 2, and unilateral hyperlucency in 1 patient. Regarding the CT results, 5 patients had mosaic attenuation, 22 had air trapping, 10 had both, and 14 had no definite airflow limitation, with only bronchial wall thickening or ground-glass opacity. In the PFT parameters, FVC, FEV1, FEV1/FVC, FEF25–75%, RV, DLCO, VA, and sRaw were statistically significantly worse in the BO group. 5 (9.8%) of the 51 patients with BO had a positive bronchodilator response > 12%. In the densitometry parameters, expiratory (EXP) MLD, MLDD, E/I volume, E/I MLD, E900, E850, E650, and E600 were all statistically significantly discriminative factors in the BO group (Table 1, Fig 2). EXP MLD (−659.0 ± 61.6 vs. −731.4 ± 69.6) and MLDD was significantly lower (129.0 ± 54.3 vs. 63.8 ± 47.2) in the patients with BO (all p < 0.001). In the BO group, the expiratory LAAs, E900 (0.4 ± 1.0 vs. 7.7 ± 11.3) and E850 (3.9 ± 9.7 vs. 22.6 ± 21.6) were higher, and the expiratory HAAs E650 (20.7 ± 10.2 vs. 10.9 ± 7.5) and E600 (26.2 ± 13.8 vs. 13.8 ± 9.2) were significantly lower than in the control group (all p < 0.001). No significant differences were found in the above-mentioned indicators during inspiration. The inspiratory MLD scatter plot by age for each group showed a distinct decrease in MLD with increasing age (Fig 3).

### Linear regression analysis

In the linear regression analysis, the LDIs showed the strongest correlation with the results of the conventional PFTs (Table 2). Specifically, FEV1 and sRaw showed significant correlations with the LDIs. MLDD (p = 0.015), E/I MLD (p = 0.018), and E/I volume (p = 0.004) showed statistically significant correlations with FEV1. The indicators that correlated with sRaw were E/I volume (p = 0.025), E850, E900, and E950 (all p < .001). The expiratory threshold parameters, including E850, E900, and E950, were significantly correlated with sRaw instead of FEV1, a conventional indicator of airflow limitation (all p values < .001). EXP MLD (p = 0.019), E600 (p < .001), and E650 (p < .001) showed statistically significant correlations with RV.

**Table 2:** Univariate and multivariate linear regression for lung density indices (LDIs) with conventional PFTs.

		Univariate				Multivariate			
		Beta	lwr	upr	*p*	Beta†	lwr	upr	*p*
EXP MLD	FEV1	0.44	0.2	0.69	< .001				
	FEV1/FVC	0.55	0.32	0.78	< .001				
	FEF25-75	0.45	0.21	0.7	< .001				
	RV	-0.65	-0.86	-0.44	< .001	-0.43	-0.78	-0.07	0.019*
	sRaw	-0.62	-0.84	-0.41	< .001	-0.27	-0.63	0.08	0.126
MLDD	FEV1	0.53	0.29	0.76	< .001	0.37	0.08	0.66	0.015*
	FEV1/FVC	0.41	0.17	0.66	0.001				
	FEF25-75	0.43	0.18	0.67	0.001				
	RV	-0.44	-0.68	-0.19	< .001				
	sRaw	-0.48	-0.72	-0.24	< .001	-0.25	-0.54	0.04	0.092
E/I MLD	FEV1	-0.53	-0.76	-0.3	< .001	-0.35	-0.64	-0.06	0.018*
	FEV1/FVC	-0.44	-0.68	-0.19	< .001				
	FEF25-75	-0.45	-0.69	-0.2	< .001				
	RV	0.48	0.24	0.72	< .001				
	sRaw	0.51	0.27	0.74	< .001	0.29	0	0.58	0.052
E/I Volume	FEV1	-0.5	-0.74	-0.27	< .001	-0.56	-0.93	-0.19	0.004**
	FEV1/FVC	-0.32	-0.58	-0.07	0.015	0.38	-0.03	0.78	0.067
	FEF25-75	-0.36	-0.61	-0.1	0.007				
	RV	0.36	0.11	0.61	0.006				
	sRaw	0.46	0.22	0.7	< .001	0.38	0.05	0.7	0.025*
E950	FEV1	-0.03	-0.05	-0.02	< .001				
	FEV1/FVC	-0.04	-0.06	-0.02	< .001				
	FEF25-75	-0.02	-0.03	-0.01	0.002				
	RV	0.01	0.01	0.02	< .001				
	sRaw	0.11	0.09	0.14	< .001	0.11	0.08	0.13	< .001**
E900	FEV1	-0.55	-0.78	-0.32	< .001				
	FEV1/FVC	-0.57	-0.79	-0.34	< .001				
	FEF25-75	-0.47	-0.71	-0.23	< .001				
	RV	0.7	0.5	0.89	< .001				
	sRaw	0.84	0.7	0.99	< .001	0.84	0.7	0.99	< .001**
E850	FEV1	-0.56	-0.78	-0.33	< .001				
	FEV1/FVC	-0.6	-0.82	-0.38	< .001				
	FEF25-75	-0.52	-0.75	-0.29	< .001	-0.16	-0.36	0.05	0.136
	RV	0.66	0.46	0.87	< .001				
	sRaw	0.76	0.59	0.94	< .001	0.68	0.47	0.89	< .001**
E650	FEV1	0.42	0.18	0.67	0.001				
	FEV1/FVC	0.46	0.22	0.7	< .001				
	FEF25-75	0.43	0.18	0.68	< .001				
	RV	-0.52	-0.76	-0.29	< .001	-0.55	-0.77	-0.32	< .001**
	sRaw	-0.44	-0.69	-0.2	< .001				
E600	FEV1	0.41	0.16	0.66	0.002				
	FEV1/FVC	0.46	0.22	0.7	< .001				
	FEF25-75	0.42	0.17	0.67	0.001				
	RV	-0.53	-0.76	-0.3	< .001	-0.55	-0.78	-0.33	< .001**
	sRaw	-0.44	-0.68	-0.19	< .001				

* p value < 0.05

** p value < 0.01

†Beta = unstandardized regression coefficient adjusted by age and gender

### Logistic regression and ROC analyses

The multivariate logistic regression analysis showed the OR of the LDIs for BO diagnosis (Table 3). The PFT parameters related to BO diagnosis were low FEV1 (OR = 0.89, p < .001), FEV1/FVC (OR = 0.89, p < .001), FEF25–75% (OR = 0.94, p < .001), and high RV (OR = 1.03, p < .001). MLDD (OR = 0.98, p < .001), E/I MLD (OR = 1.39, p < .001), E/I volume (OR = 1.11, p < .001), E900 (OR = 1.42, p = 0.036), and E600 (OR = 0.90, p < .001) were statistically significant densitometry parameters for BO diagnosis.

**Table 3:** Multivariate logistic regression of LDIs for diagnosis BO.

	Multivariate			
	OR	lcl	ucl	*p*-value
FEV1%	0.89	0.83	0.94	< .001
FEV1/FVC %	0.89	0.82	0.94	< .001
FEF25-75%	0.94	0.92	0.97	< .001
RV %	1.03	1.01	1.05	< .001
sRaw %	1.01	1	1.03	0.018
INS MLD, HU	0.99	0.98	1	0.192
EXP MLD, HU	0.98	0.97	0.99	< .001
MLDD, HU	0.98	0.96	0.99	< .001
E/I MLD %	1.39	1.15	1.67	< .001
E/I Volume %	1.11	1.03	1.19	< .001
E950%	1.01	1.00	1.02	0.006
E900%	1.42	1.02	1.96	0.036
E850%	1.54	1.16	2.04	0.003
E650%	0.87	0.81	0.94	< .001
E600%	0.90	0.85	0.95	< .001

Covariates included in multivariate model: age, gender

In the ROC analysis, the optimal cutoff values of the LDIs and PFT parameters for BO diagnosis were obtained. The sensitivity, specificity, positive predictive value, negative predictive value, and AUC for the cutoff values of the LDIs and PFT parameters are presented in Table 4. The parameters with high AUC for BO diagnosis were FEF25–75% (cutoff, 56.0%; AUC = 0.961), FEV1 (cutoff, 81%; AUC = 0.931), FEV1/FVC (cutoff, 80%; AUC = 0.897) in the conventional PFTs. In the LDIs, E900 (cutoff, 1.4%; AUC = 0.920), E950 (cutoff, 0.1%; AUC = 0.919), E850 (cutoff, 4.6%; AUC = 0.913), E/I MLD (cutoff, 86.9%; AUC = 0.887), and MLDD (109 HU; AUC = 0.867) were parameters with high AUC.

**Table 4:** Cut off, Sensitivity, specificity, and their area under the curve (AUC) with 95% CI of LDIs for diagnosis of BO.

	Cutoff	Sensitivity	Specificity	PPV	NPV	AUC	95% CI		*p*-value
FEV1%	81.0	96.6	84.2	90.3	94.1	0.931	0.86	1.00	< .001
FEV1/FVC %	80.0	89.7	84.2	89.7	84.2	0.897	0.80	0.99	< .001
FEF25-75%	56.0	93.1	100.0	100.0	905.0	0.961	0.89	1.00	< .001
RV %	132.0	65.5	94.7	95.0	64.3	0.805	0.68	0.93	< .001
sRaw %	161.0	69.0	89.5	90.9	65.4	0.807	0.68	0.93	< .001
INS MLD, HU	-841.0	55.2	84.2	84.2	55.2	0.678	0.53	0.83	< .001
EXP MLD, HU	-714.0	82.8	89.5	92.3	77.3	0.849	0.73	0.97	< .001
MLDD, HU	109.0	86.2	78.9	86.2	78.9	0.867	0.76	0.98	< .001
E/I MLD %	86.9	86.2	89.5	92.6	81.0	0.887	0.79	0.99	< .001
E/I Volume %	56.4	86.2	68.4	80.6	76.5	0.797	0.66	0.93	< .001
E950%	0.1	86.2	94.7	18.2	3.8	0.919	0.85	0.99	0.093
E900%	1.4	79.3	100.0	100.0	76.0	0.920	0.84	1.00	0.007
E850%	4.6	93.1	94.7	96.4	90.0	0.913	0.82	1.00	< .001
E650%	10.0	72.4	89.5	91.3	68.0	0.856	0.75	0.97	< .001
E600%	15.7	82.8	78.9	85.7	75.0	0.853	0.75	0.96	< .001

## Discussion

Lung densitometry is a quantitative CT method for measuring structural abnormalities of lung tissue based on the characteristics that variably attenuate X-rays. Parenchymal abnormalities can typically result in reduced attenuation in emphysema or cystic lung disease or in increased attenuation, as in pulmonary fibrosis [9]. The presence of pathological air trapping in the expiratory phase on CT scan has been defined as a parameter for detecting diseases in small airways [10]. Airflow limitation in expiratory CT scans has been suggested as the most appropriate indicator for early diagnosis of BO compared with PFTs and transbronchial biopsy [11, 12]. However, radiological detection of bronchial dilatation or definite small airway disease infrequently precedes the clinical diagnosis of patients with BO.

Previous studies performed quantitative CT analyses to detect air trapping in adult patients with COPD [13–15], asthma [16], and lung transplantation [17], and in pediatric patients with asthma [18], and BPD [19]. To the best of our knowledge, only few quantitative densitometry studies have been conducted in pediatric patients with BO [20–22]. Parameters such as density mapping, emphysema or air trapping indices, MLD, and 15th percentile density index were analyzed.

Our results suggest the possibility of quantitative pulmonary function evaluation using CT in pediatric patients with BO, as in 19 patients (23.5%) who were unable to complete spirometry measurement among the 81 patients. In our experience, in children aged 4–5 years, which is younger than the age at which spirometry can be performed, cooperation in 3-D CT to control inspiration and expiration was sufficiently possible. The LAAs range from −850 to −950 HU thresholds showed a statistically significant correlation with specific airway resistance sRaw in the multivariate analysis, indicating high OR and AUC values for BO diagnosis. In previous studies involving adult, attenuation thresholds in the range of −856 to −950 HU have been shown to be the most applicable for identifying pathological air trapping, consistent with our findings [13, 23–27].

The lung continuously grows in proportion to the body size after birth, the formation of new alveoli stops around the ages of 2–8 years [28, 29]. A recent studies reported that alveolarization continues into early adulthood [30]. Robert et al. showed that the lung structures of subjects with larger lungs differed from those with smaller lungs and that larger lungs had a thinner septum and lower parenchymal density as the air volume in the alveoli increased [31]. Lung density is expected to decrease with lung growth with increased alveolar pore volume. During the first 1–2 years of life, while the lungs grow rapidly, the lung density on CT tends to decrease rapidly [32]. In a recent study that involved children with normal lung parenchyma, MLD was significantly negatively correlated with age (r = -0.84, R2 = 0.7), which is consistent with our findings, and MLD increased by −15 HU per year [33]. However, still no consensus has been reached on the use of fixed thresholds to determine air trapping in children. Our results showed that the densitometric airflow limitation threshold was −850 to −950 HU, similar to or slightly higher than that for adults. Mocelin et al. showed that the air trapping severity and fixed PFT value through a set threshold (−950 HU) correlated in children with BO [22]. Barrera et al. suggested a threshold of −925 to −950 HU in a recent study involving adolescents with human immunodeficiency virus BO and a mean age similar to that of our cohort [20].

The methods frequently used for quantitative CT analysis of air trapping include (a) calculation of the difference or ratio of the E/I MLD and (b) measurement of the percentage of the lung area below the threshold HU during exhalation. Pathologic air trapping was defined as an attenuation difference of < 80 to 110 HU between expiration and inspiration within segmented lung parenchyma [17, 34, 35]. In our study, the cutoff of MLDD for BO diagnosis between expiration and inspiration was 109.0, which is consistent with the reports of previous studies. In the multivariate linear regression analysis, the difference (MLDD) or ratio of the E/I MLD showed a strong correlation with FEV1, and the threshold method using −850 to −950 HU showed a significant correlation with sRaw. This indicates that the LDIs and PFT parameters can be independently specific to reflect different anatomical areas that are responsible for pathological lung function.

Previous studies on E/I MLD showed stronger correlations with the spirometric airflow limitation parameters than with the density threshold-based method of air trapping [27, 36]. E/I MLD showed strong correlations with FEV1, FEV1/FVC, FEF25–75%, and FRC/TLC and the markers of respiratory morbidities [17, 36]. In our study, by contrast, expiratory −900 HU (E900) showed the highest AUC in the ROC analysis for BO diagnosis compared with the other LDIs. This was a single factor that consistently showed a significant strong correlation with the PFT parameter in various correlation analyses.

A subgroup analysis of 14 BO patients who had no definite airflow limitation findings in the radiologist’s reading showed a mean MLDD of 76.6 and E/I MLD of 94.1%, confirming that they have airflow limitation based on the cutoff criteria we derived. Therefore, CT densitometry can provide quantitative diagnostic information for the detection of early BO, where the air trapping and other representative CT features are so subtle that subjective assessment alone might fail to detect them.

Our study has several strengths. This was a study of lung densitometry in pediatric patients with BO, and it extensively investigated the correlations between various densitometry and pulmonary function parameters. Our results suggest an additional role of quantitative CT in the evaluation of pulmonary function in difficult pediatric patients with BO with poor PFT cooperation and low reliability of test results. The CT densitometry parameters presented in this study suggest the possibility of chest CT scan as an additional diagnostic test to detect airflow limitation in small-airway diseases in children rather than just as a BO-specific indicator.

The main limitation of our study is that it was a retrospective study with a small sample size. As young children undergo changes in lung volume and structures with age, the patients included in this study were likely a heterogeneous population with different lung densities. The patients included in the control group were not completely healthy children; they had other systemic diseases, such as hematologic malignancy, and thus do not represent the normal pediatric population. Although there were no pulmonary abnormalities in these patients, their test results may have been underestimated due to possible incomplete coordination of PFT or CT performance related to their poor general condition or weakness of the chest wall muscles. Additionally, it is difficult to take images in the pure inspiratory or expiratory phase in young children, and the low tidal breathing might have affected lung attenuation density. Quantitative computed tomography may have independent measurement variability between software tools, requiring standardization efforts [15]. Further study is needed to determine whether a fixed lung density threshold can be applied in pediatric BO patients of various ages.

In conclusion, lung densitometry indices using 3D chest CT scans significantly correlated with the conventional airway obstructive and resistance parameters. LAAs below the −900 HU threshold and E/I MLD ratio can be used as a quantitative marker by providing additional indications of expiratory airflow limitation for the diagnosis of children with BO.

## Supporting information

S1 File (PDF)
