# Supplementary material for: Quantitative CT lung densitometry as an obstructive marker for the diagnosis of bronchiolitis obliterans in children
Source: PLoS One. 2022 Jul 7;17(7):e0271135. doi: 10.1371/journal.pone.0271135 (PMC9262182; doi:10.1371/journal.pone.0271135)
Supplement: S1 File — (PDF) [file pone.0271135.s001.pdf]

Patient #	Diagnosis	MLDD	EI_MLD	EI_Volume	I900	I850	I650
1	NO	144	0.813472	0.503813	3.2	22.8	4.3
2	BO_HSCT	60	0.92665	0.729391	10.1	54.3	3.2
3	BO_HSCT	179	0.789659	0.440732	36.6	75.2	2.5
4	BO_HSCT	59	0.930178	0.709038	39.1	69.3	2.4
5	BO_PIBO	-1	1.001513	1	0.5	15	4.2
6	BO_HSCT	108	0.868613	0.626782	15.2	50.3	3.7
7	BO_HSCT	100	0.882904	0.577194	54.6	81.4	1.9
8	NO	159	0.809353	0.458358	20.2	65	2.6
9	BO_HSCT	83	0.905682	0.591976	59.3	83.7	1.7
10	BO_HSCT	37	0.955847	0.809848	47.3	75	2.7
11	NO	94	0.862573	0.729204	0.1	1	10.8
12	BO_HSCT	89	0.897701	0.65344	45.8	79.6	1.9
13	BO_HSCT	42	0.951445	0.717644	48.4	77.9	2.1
14	BO_HSCT	39	0.953181	0.772564	24.5	61.8	2.7
15	BO_HSCT	134	0.845087	0.477252	54.6	81.4	1.9
16	BO_PIBO	98	0.883472	0.513065	25	67.4	2.5
17	BO_PIBO	NA	NA	NA	0.5	15	4.2
18	BO_HSCT	NA	NA	NA	36.6	65.6	3.1
19	BO_HSCT	33	0.961085	0.796165	47.3	75	16.2
20	NO	107	0.854817	0.741717	0.5	6.6	5.7
21	NO	180	0.784173	0.414767	26.6	71.7	2.6
22	BO_PIBO	50	0.936061	0.78284	10.3	31.3	4.5
23	BO_HSCT	109	0.871614	0.564265	35.6	71.8	2.5
24	NO	206	0.762673	0.362743	51.2	81.7	1.9
25	NO	170	0.78882	0.490055	5.4	39.5	3.1
26	NO	38	0.955347	0.791667	36.5	78.3	2.1
27	BO_HSCT	74	0.894134	0.730857	0	1.1	9.2
28	NO	106	0.855978	0.644522	0.2	4.9	6.6
29	BO_PIBO	56	0.923288	0.802603	5.4	19.6	7.4
30	BO_HSCT	108	0.868613	0.597165	18	53	3.4
31	NO	87	0.897406	0.614859	29.7	75.7	2.2
32	BO_HSCT	51	0.92093	0.214565	5.5	15.6	11.2
33	BO_HSCT	13	0.985109	0.887758	56.9	78.6	2.4
34	BO_HSCT	16	0.980975	0.881481	2.6	18.7	4.7
35	BO_HSCT	104	0.863338	0.645714	2.6	3.7	4.7
36	BO_HSCT	66	0.912117	0.756098	3.2	16.4	5.4
37	NO	145	0.799724	0.644554	5.2	6.4	8.2
38	BO_HSCT	64	0.9201	0.678924	8.9	36.8	3.5
39	BO_HSCT	-34	1.053712	1.126292	0	0.5	23.3
40	BO_HSCT	2	0.997319	0.988855	3.9	17.5	6.7
41	NO	170	0.795181	0.443044	21.6	55.7	2.8
42	BO_HSCT	69	0.920507	0.670792	49.6	77.5	2.2

43	BO_HSCT	13	0.984866	0.898642	49.3	75.6	2.7
44	NO	182	0.786134	0.399547	30.4	74.1	2.4
45	NO	154	0.814234	0.496279	18.6	60.8	2.8
46	NO	19	0.969106	0.936057	0	0	25.7
47	BO_HSCT	17	0.977922	0.929088	2.9	24.9	4.5
48	BO_HSCT	8	0.990476	0.947084	30.8	71.1	22.7
49	NO	112	0.864242	0.585279	10.5	56.1	2.7
50	BO_HSCT	15	0.981685	0.920598	17.5	62	3.1
51	BO_HSCT	135	0.815825	0.544756	14.3	34.6	7.7
52	NO	136	0.826087	0.562975	3.8	27.8	4.2
53	BO_HSCT	13	0.981586	1.018462	0.6	5.9	11.9
54	NO	140	0.826947	0.551563	14.6	43.5	3.3
55	BO_HSCT	37	0.956265	0.85828	49.1	70	3.6
56	NO	121	0.849502	0.613773	4.4	40.5	3.2
57	BO_HSCT	50	0.938042	0.766144	20.1	51.3	1.69
58	BO_HSCT	10	0.984252	0.966272	0.1	0.6	24.7
59	BO_HSCT	-10	1.013928	0.990903	0.5	4.5	10.1
60	NO	182	0.782816	0.443469	28.5	68.3	2.9
61	BO_PIBO	46	0.934566	0.849682	2.8	16.8	12.6
62	NO	41	0.928696	0.831418	0	0	41.9
63	NO	52	0.932203	0.82227	1	15.8	4.2
64	NO	147	0.821385	0.512215	11.2	56.3	2.9
65	BO_HSCT	109	0.871614	0.564265	35.6	71.8	2.5
66	BO_HSCT	206	0.762673	0.362743	51.2	81.7	1.9
67	NO	170	0.78882	0.490055	5.4	39.5	3.1
68	BO_HSCT	74	0.894134	0.730857	0	1.1	9.2
69	BO_PIBO	56	0.923288	0.802603	5.4	19.6	7.4
70	BO_HSCT	108	0.868613	0.597165	18	53	3.4
71	NO	87	0.897406	0.614859	29.7	75.7	2.2
72	NO	69	0.893519	0.686435	0.7	3.7	15.8
73	BO_HSCT	51	0.92598	0.845192	0.4	3.3	10.9
74	NO	80	0.903382	0.628758	15.1	60.3	2.5
75	BO_HSCT	54	0.935943	0.727113	32.8	68.2	2.6
76	NO	179	0.768135	0.480659	3	20.3	2.9
77	BO_HSCT	125	0.841371	0.606368	9.2	36	3.7
78	NO	252	0.706294	0.312918	40.6	77.8	2.2
79	NO	140	0.833135	0.475543	23	70.2	2.6
80	BO_HSCT	72	0.916955	0.603167	54.2	79.5	2.2
81	BO_HSCT	97	0.885613	0.639454	30.6	70	2.4

I600	E900	E850	E650	E600	INS_V	INS_MLD	IV950
5.5	0.1	0.9	27.8	34.5	3803	-772	0.2
4.2	0.5	12	5.8	7.4	3433	-818	0.1
3.3	0.3	4.6	17.7	23.1	3442	-851	1
3.3	11.1	39.1	4	5.4	2069	-845	3.6
5.7	NA	NA	NA	NA	751	-661	0
4.8	1.5	9.8	10	12.4	1613	-822	1.1
2.7	2.7	12.4	8	10	4262	-854	4.8
3.4	0.2	1.7	17.1	20.7	3434	-834	1
2.2	7.8	34.4	3.3	4.3	5409	-880	11.1
3.8	22.4	59.4	3.4	4.7	1909	-838	5.3
13.1	0	0.1	38.5	48.1	1130	-684	0
2.5	6.4	25.2	4.2	5.3	4637	-870	6.2
2.7	21.6	56.2	3.3	4.3	3701	-865	5.2
3.5	8.3	34.8	3.7	4.8	3346	-833	1.6
2.7	2.7	12.4	8	10	4440	-865	4.8
3.3	25.9	41.6	17	22	3253	-841	2.2
5.7	NA	NA	NA	NA	1314	-765	0
4.3	NA	NA	NA	NA	980	-831	3.2
17.3	22.4	59.4	3.4	4.7	1982	-848	5.3
7.2	0.1	0.7	28.2	34.6	2505	-737	0
3.6	0	0.9	21.7	26.3	2763	-834	0.6
5.8	5	19.7	12	14.4	2634	-782	0.9
3.3	1.9	11.9	7.7	9.6	2373	-849	2.7
2.5	0.3	2.6	18.1	23	5395	-868	4.2
4	0.1	0.6	25.2	31	3067	-805	0.2
2.9	5.7	52.1	2.7	3.7	2664	-851	0.7
11.5	0	0.2	27	32.8	2181	-699	0
8.3	0	0.2	26.8	33.3	1716	-736	0
9.6	3.5	15.8	21.9	26.8	922	-730	0.3
4.4	0.28	8.78	12.8	15.7	1693	-822	1.4
2.9	0.9	12.6	4.7	6	5707	-848	0.4
15.7	1.1	1.7	21.2	27.1	2046	-645	2.1
3.2	48.8	75.4	2.7	3.5	3831	-873	14.6
6.3	0.4	3.2	13.9	17.7	1350	-841	0.2
6.6	0.4	3.2	19.9	23.7	1225	-761	0.2
7.2	1.2	7.3	14.1	17.2	1230	-751	0.2
10.2	0	0	35.3	44.9	1010	-724	1
4.6	1.6	11.3	7.8	9.6	2529	-801	0.7
29	0	0.7	17.1	20.9	871	-633	0
8.8	3.4	16.4	6.3	8.4	987	-746	0.4
3.7	0.2	1.3	18.9	23.4	2063	-830	3.5
2.9	11.6	43	4	5.2	3232	-868	9.5

3.5	40.5	71.4	3.1	4.1	3828	-859	6.3
3.1	0.1	1.7	18.1	22.8	2648	-851	1.6
3.7	0.3	2.6	17.7	21.6	3897	-829	1
32.4	0	0	29.6	37.8	563	-615	0
6.1	1.2	15.6	5.1	6.8	1382	-770	0.1
66.6	22.7	66.6	2.7	3.7	1852	-840	1.1
3.5	0.1	1.9	10.2	12.3	3383	-825	0.3
4.3	11.3	50.3	3.4	4.7	1738	-819	0.5
10.5	3.6	7	8.9	15.7	2603	-733	3.6
5.4	0.1	0.7	22.1	27.2	2501	-782	0.2
14.4	0.6	6.2	13.3	16.4	975	-706	0
4.3	0.7	1	17.9	22.3	2783	-809	2.1
4.8	34.4	59.2	4.3	5.9	3895	-846	9.6
4.2	0	0.4	14.4	17.4	3151	-804	0
2.89	7.3	27.3	6.3	8.2	1595	-807	2.9
30.9	0	0.3	28.1	35.2	1097	-635	0
12.7	0.6	6.4	8.9	11.2	1429	-718	0.1
3.8	0.1	1.9	19.7	24.6	1822	-838	1.8
15.3	2.7	17.4	19.9	27.7	785	-703	0.1
52.7	0	0	43.7	63.3	522	-575	0
5.6	0.2	3.4	7.6	9.7	1868	-767	0
3.8	0.1	2.2	18.9	22.2	2497	-823	0.3
3.3	1.9	11.9	7.7	9.6	2373	-849	2.7
2.5	0.3	2.6	18.1	23	5395	-868	4.2
4	0.1	0.6	25.2	31	3067	-805	0.2
11.5	0	0.2	27	32.8	2181	-699	0
9.6	3.5	15.8	21.9	26.8	922	-730	0.3
4.4	1.9	10.4	12.8	15.7	1693	-822	1.4
2.9	0.9	12.6	4.7	6	5707	-848	0.4
19.7	0.1	0.6	29.3	37.9	1585	-648	0.1
13.9	0.2	1.7	20.7	25.2	1040	-689	0
3.3	1.2	11	5.8	7.3	4889	-828	0.5
3.4	7.1	33.6	4.3	5.5	4555	-843	1.9
4.2	0	0.3	29.4	39.4	2792	-772	0.2
5	1.4	5.2	18.3	21.9	1382	-788	0.6
2.9	0	0.3	28.9	38.5	4033	-858	2
3.4	0.3	3.5	12.4	15	3087	-839	0.9
2.9	11.7	39.4	4.3	5.6	4105	-867	8.3
3.1	3	16.5	6.2	7.8	3589	-848	2.4

IV900	IV850	IV650	IV600	IV550	EXP_V	EXP_MLD	EXP_SD
3	19.6	2.5	1.8	1.2	1916	-628	160
10	44.2	1.8	1.4	1	2504	-758	128
35.6	38.6	1.4	1.1	0.8	1517	-672	168
35.5	30.2	1.3	1.1	0.9	1467	-786	148
0.5	14.5	2.4	1.8	1.5	751	-662	156
14.1	35.1	2.1	1.6	1.1	1011	-714	150
49.8	26.8	1	0.9	0.8	2460	-754	153
19.2	44.8	1.4	1.2	0.8	1574	-675	150
48.2	24.4	0.9	0.8	0.5	3202	-797	136
42	27.7	1.5	1.2	1.1	1546	-801	158
0.1	0.9	7.2	3.6	2.3	824	-590	142
39.6	33.8	1	0.9	0.6	3030	-781	132
43.2	29.5	1.2	0.9	0.6	2656	-823	134
22.9	37.3	1.5	1.2	0.8	2585	-794	141
49.8	26.8	1	0.9	0.8	2119	-731	150
22.8	42.4	1.4	1.1	0.8	1669	-743	190
0.5	14.5	2.4	1.8	1.5	NA	NA	NA
33.4	29	1.7	1.4	1.2	NA	NA	NA
42	27.7	15	1.2	1.1	1578	-815	158
0.5	6.1	3.4	2.3	1.5	1858	-630	152
26	45.1	1.4	1.2	1	1146	-654	145
9.4	21	2.6	1.9	1.3	2062	-732	157
32.9	36.2	1.4	1.1	0.8	1339	-740	138
47	30.5	1	0.9	0.6	1957	-662	167
5.2	34.1	1.7	1.4	0.9	1503	-635	152
35.8	41.8	1.1	1	0.8	2109	-813	132
0	1.1	5.6	3.6	2.3	1594	-625	155
0.2	4.7	4	2.6	1.7	1106	-630	142
5.1	14.2	4.5	2.9	2.2	740	-674	173
16.6	35	1.9	1.5	1	1011	-714	150
29.3	46	1.2	1	0.7	3509	-761	138
3.4	10.1	5.9	5.3	4.5	439	-594	200
42.3	21.7	1.3	1.1	0.8	3401	-860	139
2.4	16.1	2.7	2	1.6	1190	-825	158
2.4	1.1	2.7	2	1.9	791	-657	152
3	13.2	3.1	2.3	1.8	930	-685	150
4.2	1.2	5.2	3	2	651	-579	142
8.2	27.9	2	1.5	1.1	1717	-737	174
0	0.5	13.3	10	5.7	981	-667	136
3.5	13.6	3.9	2.8	2.1	976	-744	148
18.1	34.1	1.6	1.2	0.9	914	-660	148
40.1	27.9	1.2	1	0.7	2168	-799	145

43	26.3	1.5	1.2	0.8	3440	-846	140
28.8	43.7	1.3	1.1	0.7	1058	-669	147
17.6	42.2	1.6	1.2	0.9	1934	-675	150
0	0	15.5	10.2	6.7	527	-596	146
2.8	22	2.6	1.9	1.6	1284	-753	153
29.7	40.3	0.8	21.9	43.9	1754	-832	134
10.2	45.6	1.5	1.2	0.8	1980	-713	129
17	44.5	1.7	1.4	1.2	1600	-804	146
10.7	20.3	4.2	3.5	2.8	1418	-598	233
3.6	24	2.4	1.8	1.2	1408	-646	153
0.6	5.3	7.7	4.2	2.5	993	-693	154
12.5	28.9	1.9	1.4	1	1535	-669	160
39.5	20.9	2	1.6	1.2	3343	-809	184
4.4	36.1	1.8	1.4	1	1934	-683	138
17.2	31.2	0.19	1.5	1.2	1222	-757	166
0.1	0.5	14.7	10	6.2	1060	-625	149
0.4	4	6.1	4	2.6	1416	-728	141
26.7	39.8	1.6	1.3	0.9	808	-656	161
2.7	14	8.3	4.3	2.7	667	-657	200
0	0	23.5	18.4	10.8	434	-534	132
1	14.8	2.4	1.8	1.4	1536	-715	144
10.9	45.1	1.6	1.3	0.9	1279	-676	141
32.9	36.2	1.4	1.1	0.8	1339	-740	138
47	30.5	1	0.9	0.6	1957	-662	167
5.2	34.1	1.7	1.4	0.9	1503	-635	152
0	1.1	5.6	3.6	2.3	1594	-625	155
5.1	14.2	4.5	2.9	2.2	740	-674	173
16.6	35	1.9	1.5	1	1011	-714	150
29.3	46	1.2	1	0.7	3509	-761	138
0.6	3	9.7	6.1	3.9	1088	-579	189
0.4	2.9	6.5	4.4	3	879	-638	164
14.6	45.2	1.4	1.1	0.8	3074	-748	138
30.9	35.4	1.4	1.2	0.8	3312	-789	137
2.8	17.3	2.7	0.2	1.3	1342	-593	158
8.6	26.8	2.1	1.6	1.3	838	-663	159
38.6	37.2	1.2	1	0.7	1262	-606	154
22.1	47.2	1.4	1.2	0.8	1468	-699	146
45.9	25.3	1.2	1	0.7	2476	-795	148
28.2	39.4	1.3	1.1	0.7	2295	-751	140

EV950	EV900	EV850	EV650	EV600	FVC	FEV1	FEV1_FVC
0	0.1	0.8	16.4	11.4	93	82	84
0	0.5	11.5	3.4	2.4	90	91	97
0	0.3	4.3	9.8	7.9	78	56	69
0.3	10.8	28	2.3	1.7	77	47	55
NA	NA	NA	NA	NA	NA	NA	NA
0.1	1.4	8.3	6.2	3.8	68	50	54
0.3	2.4	9.7	4.9	3.1	82	63	70
0	0.2	1.5	10.7	6.4	100	97	92
0.6	7.2	26.6	1.9	1.4	102	81	97
2.3	20.1	37	1.9	1.5	86	80	83
0	0	0.1	21.7	16.8	66	65	90
0.6	5.8	18.8	2.5	1.7	59	51	80
1.3	20.3	34.6	1.9	1.4	102	34	31
0.4	7.9	26.5	2.1	1.6	93	52	54
0.3	2.4	9.7	4.9	3.1	84	70	77
3.3	22.6	15.7	9.5	7.5	83	72	78
NA	NA	NA	NA	NA	NA	NA	NA
NA	NA	NA	NA	NA	NA	NA	NA
2.3	20.1	37	1.9	1.5	NA	NA	NA
0	0.1	0.6	17.1	11.1	93	83	82
0	0	0.9	13.6	8.1	96	94	89
0.4	4.6	14.7	7.6	4.4	78	62	72
0.1	1.8	10	4.7	3	83	44	48
0	0.3	2.3	10.5	7.6	NA	NA	NA
0	0.1	0.5	15.3	9.9	100	82	76
0.1	5.6	46.4	1.5	1.2	80	75	87
0	0	0.2	16.4	10.6	83	69	76
0	0	0.2	16.1	10.7	102	95	85
0.2	3.3	12.3	13.4	8.5	NA	NA	NA
0.1	0.18	8.5	7.9	4.9	106	72	60
0	0.9	11.7	2.7	2	117	112	88
0.5	0.6	0.6	12.6	8.6	NA	NA	NA
8.9	39.9	26.6	1.5	1.2	43	18	39
0	0.4	2.8	13.4	0.5	NA	NA	NA
0	0.4	2.8	13.4	6.5	58	64	99
0	1.2	6.1	9.4	4.7	83	73	78
0	0	0	20.5	14.8	NA	NA	NA
0.1	1.5	9.7	4.8	3	86	45	48
0	0	0.7	11	6.1	NA	NA	NA
0.2	3.2	13	3.7	2.6	104	91	74
0	0.2	1.1	11.7	7.2	92	88	87
0.8	10.8	31.4	2.3	1.7	101	38	34

4	36.5	30.9	1.7	1.4	43	18	39
0	0.1	1.6	10.8	7.3	97	85	83
0	0.3	2.3	11	6.7	75	66	85
0	0	0	17.6	12	NA	NA	NA
0	1.2	14.4	2.9	2.2	31	25	73
0.8	21.9	43.9	1.5	1.2	NA	NA	NA
0	0.1	1.8	6.5	3.7	97	101	96
0.5	10.8	39	1.9	1.5	25	19	70
2.1	1.5	3.4	8.7	0.2	32	26	70
0	0.1	0.6	13.4	8.7	101	97	88
0	0.6	5.6	8.5	4.8	91	100	94
0.1	0.6	0.3	10.8	7.1	111	86	73
6.9	27.5	24.8	2.4	1.9	70	29	39
0	0	0.4	9.1	5.3	80	69	83
1	6.3	20	3.8	2.5	NA	NA	NA
0	0	0.3	16.4	11.7	41	38	87
0.1	0.5	5.8	5.3	3.6	47	43	74
0	0.1	1.8	11.7	8	91	93	92
0.1	2.6	14.7	9.8	10.1	NA	NA	NA
0	0	0	18.9	24.8	NA	NA	NA
0	0.2	3.2	4.7	2.9	84	89	100
0	0.1	2.1	12.6	6.3	99	95	87
0.1	1.8	10	4.7	3	83	44	48
0	0.3	2.3	10.5	7.6	NA	NA	NA
0	0.1	0.5	15.3	9.9	100	84	76
0	0	0.2	16.4	10.6	83	69	76
0.2	3.3	12.3	13.4	8.5	NA	NA	NA
0.1	1.8	8.5	7.9	4.9	106	72	60
0	0.9	11.7	2.7	2	117	112	88
0	0.1	0.5	16.6	12.7	NA	NA	NA
0	0.2	1.5	13.4	7.3	27	27	92
0.1	1.1	9.8	3.5	2.3	NA	NA	NA
0.4	6.7	26.5	2.5	1.8	78	33	39
0	0	0.3	16	13.4	107	100	86
0.1	1.3	3.8	12.3	6	83	70	77
0	0	0.3	15.8	13.1	92	82	85
0	0.3	3.2	7.8	4.6	98	89	86
1.2	10.5	27.7	2.4	1.9	76	44	55
0.1	2.9	13.5	3.8	2.4	57	43	70

FEF25_75	TLC	RV	DLCO	DLCO	VA	Raw	Raw_p
75	87	75	16.9	68	3.95	2.02	77
123	88	67	19	75	3.75	2.19	84
30	88	128	12.4	48	3.17	4.74	183
19	112	210	9.4	73	1.55	7.47	154
NA	NA	NA	NA	NA	NA	NA	NA
18	113	147	7.4	65	1.01	5.98	105
36	96	143	19	76	3.94	2	76
102	96	93	20	88	3.81	2.38	85
56	114	167	18	67	4.78	2.41	97
59	79	53	7.2	54	1.29	3.35	73
90	63	31	7.4	53	1.4	4.66	107
40	89	209	22	71	4.13	2.05	90
11	132	223	11.1	73	2.48	8.21	208
20	103	151	17.5	74	3.6	2.81	103
51	107	190	23.4	94	4.42	1.87	71
53	105	188	15.4	89	2.32	4.68	134
NA	NA	NA	NA	NA	NA	NA	NA
NA	NA	NA	NA	NA	NA	NA	NA
NA	NA	NA	NA	NA	NA	NA	NA
72	80	37	24.3	102	3.9	3.29	122
91	94	91	14.7	89	2.5	3.08	84
39	94	152	15.3	75	2.48	5.44	179
17	118	235	13	100	1.93	7.39	155
NA	NA	NA	NA	NA	NA	NA	NA
61	100	104	24.5	104	4.21	4.4	161
70	102	92	NA	NA	NA	7.86	208
49	87	108	8.1	45	2.59	2.65	79
89	91	59	16.1	93	2.87	3.99	114
NA	NA	NA	NA	NA	NA	NA	NA
25	127	106	11.1	94	1.78	4.46	82
114	112	84	26	90	6	2.13	90
NA	NA	NA	NA	NA	NA	NA	NA
7	115	372	5.5	28	2.08	15.33	492
NA	NA	NA	NA	NA	NA	NA	NA
164	NA	NA	4.9	43	1.12	NA	NA
47	108	178	8	73	1.14	6.46	112
NA	NA	NA	NA	NA	NA	NA	NA
15	84	82	17	86	2.92	4.89	157
NA	NA	NA	NA	NA	NA	NA	NA
45	NA	NA	NA	NA	NA	NA	NA
92	91	76	11.2	68	2.88	2.44	66
11	157	331	8.5	64	1.91	8.18	182

7	115	372	5.5	28	2.08	15.33	492
83	84	47	10.3	54	2.92	2.54	80
58	94	169	16.8	67	3.65	2.77	106
NA	NA	NA	NA	NA	NA	NA	NA
15	NA	NA	NA	NA	NA	NA	NA
NA	NA	NA	NA	NA	NA	NA	NA
116	99	111	24.3	88	4.93	1.88	77
11	NA	NA	NA	NA	NA	NA	NA
15	34	42	5.7	17	2.15	2.71	242
101	107	129	16	77	3.49	3.06	102
91	NA	NA	NA	NA	NA	NA	NA
58	110	119	12.7	62	3.81	3.02	99
9	120	292	11.7	60	2.73	8.93	284
59	90	109	16.6	66	3.55	2.43	92
NA	NA	NA	NA	NA	NA	NA	NA
39	NA	NA	NA	NA	NA	NA	NA
23	65	132	12.7	81	2.11	7.99	208
96	104	138	9.2	71	2.02	3.75	79
NA	NA	NA	NA	NA	NA	NA	NA
NA	NA	NA	NA	NA	NA	NA	NA
115	90	116	11.4	67	2.67	3.9	110
90	97	94	13.1	81	2.59	4.45	119
17	118	235	13	100	1.93	7.39	155
NA	NA	NA	NA	NA	NA	NA	NA
61	100	104	24.5	104	4.21	4.4	161
49	87	108	8.1	45	2.59	2.65	79
NA	NA	NA	NA	NA	NA	NA	NA
25	127	202	11.1	94	1.78	4.46	82
114	112	84	26	90	6	2.13	90
NA	NA	NA	NA	NA	NA	NA	NA
54	52	131	NA	NA	NA	2.37	74
NA	NA	NA	NA	NA	NA	NA	NA
12	106	219	20.5	65	3.51	5.53	242
103	93	45	19.9	71	4.89	2.2	91
46	90	109	9.7	79	1.45	4.7	94
74	90	90	15.3	65	3.73	3.3	120
83	94	87	13.9	66	3.47	2.26	76
16	76	83	13.9	53	3.25	2.83	110
28	78	164	19.5	56	3.5	2.9	260

Gaw	Gaw_p	sRaw	sRaw_p	sGaw	sGaw_p
0.495	364	6.33	108	0.158	60
0.457	338	6.78	116	0.148	56
0.211	157	12.47	212	8	30
0.134	72	15.19	319	0.066	53
NA	NA	NA	NA	NA	NA
0.167	85	8.13	187	123	-70
0.501	370	7.1	121	0.141	62
0.45	294	6.74	117	0.148	57
0.415	320	10.16	172	0.098	43
0.299	163	3.86	79	0.259	224
0.214	119	5.91	118	0.169	70
0.488	410	8.24	137	0.121	52
0.122	70	25.6	491	0.039	16
0.357	256	9.35	161	0.107	40
0.536	396	7.53	129	0.133	58
0.214	131	11.23	207	0.089	48
NA	NA	NA	NA	NA	NA
NA	NA	NA	NA	NA	NA
NA	NA	NA	NA	NA	NA
0.304	219	6.43	111	0.155	74
0.325	195	6.04	113	0.166	91
0.184	122	15.28	270	0.065	33
0.135	73	15.54	323	0.064	68
NA	NA	NA	NA	NA	NA
0.227	162	10.54	182	0.095	44
0.127	75	17.51	331	0.057	23
0.378	236	5.86	107	0.171	88
0.251	153	6.75	124	0.148	79
NA	NA	NA	NA	NA	NA
0.224	116	7.22	162	0.139	-143
0.47	380	8.35	140	0.12	54
NA	NA	NA	NA	NA	NA
0.065	43	61.82	1101	0.016	8
NA	NA	NA	NA	NA	NA
NA	NA	NA	NA	NA	NA
0.155		6.96	161	0.144	74
NA	NA	NA	NA	NA	NA
0.205	164	15.61	278	0.064	32
NA	NA	NA	NA	NA	NA
NA	NA	NA	NA	NA	NA
0.409	244	5	94	0.2	115
0.122	NA	25.16	509	0.04	17

0.065	43	61.82	1101	0.016	8
0.393	254	5.59	100	0.179	69
0.361	267	8.59	147	0.116	44
NA	NA	NA	NA	NA	NA
NA	NA	NA	NA	NA	NA
NA	NA	NA	NA	NA	NA
0.532	418	6.07	102	0.165	72
NA	NA	NA	NA	NA	NA
0.37	39	5.51	126	0.181	80
0.37	219	6.5	115	0.154	77
NA	NA	NA	NA	NA	NA
0.331	220	8.57	152	0.117	45
0.112	73	34.92	623	0.029	11
0.412	303	6.99	119	0.143	54
NA	NA	NA	NA	NA	NA
NA	NA	NA	NA	NA	NA
0.125	73	10.73	204	0.093	38
0.266	NA	5.61	117	0.178	244
NA	NA	NA	NA	NA	NA
NA	NA	NA	NA	NA	NA
0.256	156	7.53	139	0.133	52
0.225	133	7.27	137	0.138	83
0.135	73	15.54	323	0.064	68
NA	NA	NA	NA	NA	NA
0.227	162	10.54	182	0.095	44
0.378	236	5.86	107	0.171	88
NA	NA	NA	NA	NA	NA
0.224	116	7.22	162	0.139	-143
0.47	380	8.35	140	0.12	54
NA	NA	NA	NA	NA	NA
0.422	270	3.29	59	0.304	118
NA	NA	NA	NA	NA	NA
0.181	151	25.17	418	0.04	17
0.454	360	5.11	86	0.196	89
0.213	113	6.79	144	0.147	66
0.303	216	9.03	156	0.111	42
0.443	298	5.63	99	0.177	68
0.353	265	9.87	168	0.101	38
0.344	37	8.84	201	0.113	50
